# Effect of Post-Annealing on Barrier Modulations in Pd/IGZO/SiO_2_/p^+^-Si Memristors

**DOI:** 10.3390/nano12203582

**Published:** 2022-10-13

**Authors:** Donguk Kim, Hee Jun Lee, Tae Jun Yang, Woo Sik Choi, Changwook Kim, Sung-Jin Choi, Jong-Ho Bae, Dong Myong Kim, Sungjun Kim, Dae Hwan Kim

**Affiliations:** 1 School of Electrical Engineering, Kookmin University, Seoul 02707, Korea; 2 Division of Electronics and Electrical Engineering, Dongguk University, Seoul 04620, Korea

**Keywords:** neuromorphic system, synaptic device, annealing, indium gallium zinc oxide, neuromorphic simulation

## Abstract

In this article, we study the post-annealing effect on the synaptic characteristics in Pd/IGZO/SiO_2_/p^+^-Si memristor devices. The O-H bond in IGZO films affects the switching characteristics that can be controlled by the annealing process. We propose a switching model based on using a native oxide as the Schottky barrier. The barrier height is extracted by the conduction mechanism of thermionic emission in samples with different annealing temperatures. Additionally, the change in conductance is explained by an energy band diagram including trap models. The activation energy is obtained by the depression curve of the samples with different annealing temperatures to better understand the switching mechanism. Moreover, our results reveal that the annealing temperature and retention can affect the linearity of potentiation and depression. Finally, we investigate the effect of the annealing temperature on the recognition rate of MNIST in the proposed neural network.

## 1. Introduction

As a new system to overcome the data processing speed and energy consumption of the von Neumann computing structure, the neuromorphic system that mimics the human brain is attracting much attention [1,2,3,4,5,6,7,8,9]. Neuromorphic computing employs a biological neural network system for efficient information processing with low energy consumption. Neurons using CMOS and synapses using memory devices are building blocks in neuromorphic systems. It is essential to develop a high-performance non-volatile memory that can be implemented as hardware. The performance indicators of non-volatile memory are linearity [10,11,12,13], retention [14,15,16], endurance [17,18,19], the number of conductance states [20,21,22,23], power consumption, [24,25,26,27] and device variation [28,29,30,31,32]. Improvement of synaptic characteristics by optimization of the device can simply enhance the performance of neuromorphic systems. The annealing process is one powerful way. Several studies are still in progress, designed to improve these indicators at the device level through process optimization. In this paper, the characteristics of memristors using IGZO as a resistive switching layer are analyzed by the process conditions to implement a hardware-based neuromorphic system. IGZO is a promising material for the design of next-generation intelligent semiconductors. Power consumption is efficient because of the low leakage current [33,34,35,36], and large-area processing is possible owing to high uniformity [37]. In addition, low-temperature processing is possible, and there are no restrictions on the substrate, which is advantageous for flexible device manufacturing [38], and the process compatibility with the back-end-of-line (BEOL) processing is good [39,40,41]. Furthermore, IGZO is a suitable material for non-volatile memory devices. Oxidation and reduction reactions occur inside IGZO depending on the external bias, which changes the resistance of IGZO; thus, IGZO is used as a memory device by utilizing these characteristics [42,43]. In fact, there have already been studies on various memory devices using IGZO [44,45,46,47,48,49]. In particular, the p^+^Si-based IGZO memristors introduced in this paper show high retention and endurance characteristics.

In previous studies, the resistive characteristics of memristors depending on the oxygen content of IGZO were analyzed to optimize the process of p^+^Si-based IGZO memristors [42,50]. The higher the oxygen content in IGZO, the better the linearity and the lower the power consumption. However, when the oxygen content of IGZO is high, the conductance state is reduced because the overall current is lowered. In this study, a post-annealing process was performed to retain the advantages of IGZO when the oxygen content is high and to compensate for the disadvantages. Post-annealing is a technique often used to accelerate the diffusion of oxygen and hydrogen inside IGZO and to improve the hysteresis window, mobility, on/off ratio, and uniformity [51,52,53]. We clearly understand the operating mechanism of the p^+^Si-based IGZO memristors, and based on this, we conducted characterizations, such as a number of conductance states, linearity, retention, and endurance, depending on the post-annealing process. Furthermore, the post-annealing process conditions were optimized for a deep neural network (DNN), which is one neuromorphic system, to achieve optimal performance.

## 2. Experimental Setup

**Figure 1:**
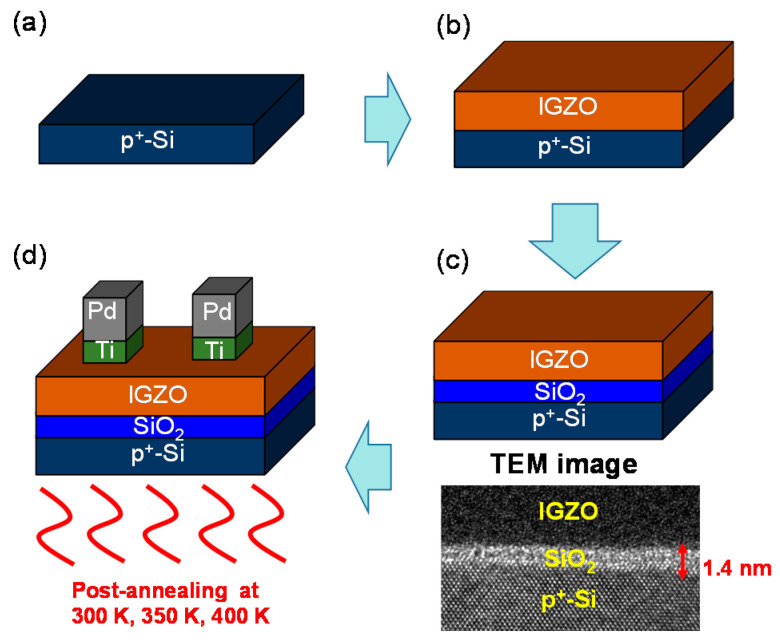
Process flow of Pd/IGZO/SiO_2_/p^+^-Si memristors: (**a**) p^+^-Si formation; (**b**) IGZO deposition by RF sputtering system; (**c**) Native oxide formation and TEM image of IGZO/SiO_2_/p^+^-Si layers; (**d**) Pd/Ti deposition by e-beam evaporation.

Pd/IGZO/SiO_2_/p^+^-Si memristors were prepared as shown in Figure 1a–d. Initial cleaning of the p-type Si wafer with a boron doping concentration of 2 × 10^19^ cm^−3^ was performed (Figure 1a). The p-type Si wafer functioned as the bottom electrode (BE) of the memristor. An 80-nanometer-thick IGZO film as a switching layer was deposited at 150 W at room temperature with a radiofrequency (RF) sputtering system. At this time, the Ar and O_2_ flow rates were 3 sccm and 2 sccm, respectively (Figure 1b). After IGZO deposition, an approximately 1.4-nanometer-thick SiO_2_ layer was formed as a native oxide between the p^+^-Si and IGZO layers, which was confirmed by the transmission electron microscopy (TEM) image (Figure 1c). Next, Ti and Pd were deposited by e-beam evaporation as an adhesion layer and a top electrode, respectively (Figure 1d). The deposition rate was slowed to 0.5 Å/s to stabilize the interface characteristics and keep the atomic matrix dense. In addition, Ti and Pd patterning was conducted using a shadow mask with a rectangular pattern, with the dimensions of 100 μm × 300 μm. After, post-annealing was performed in an oven at 300 K, 350 K, and 400 K in an air environment without partial pressure gases for 1 h.

## 3. Results and Discussion

**Figure 2:**
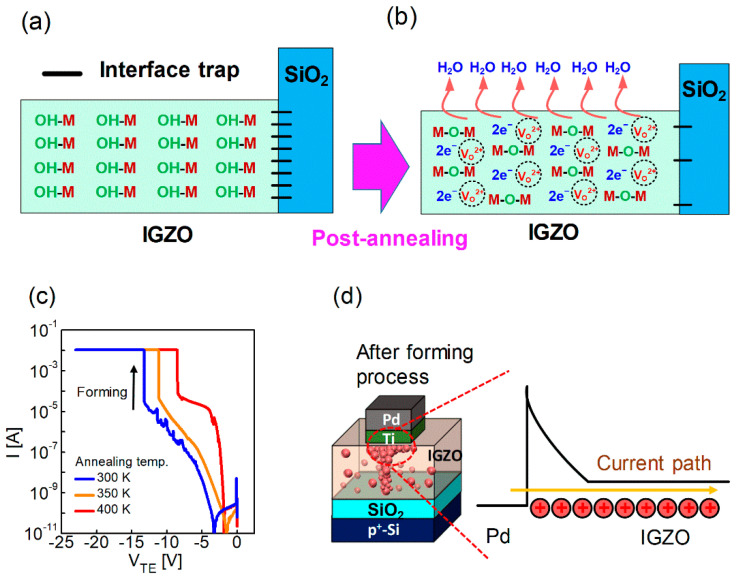
Illustration of O-H bond, H_2_O, oxygen vacancy, and electrons in IGZO and interface trap between the IGZO and SiO_2_ layers (**a**) before and (**b**) after annealing; (**c**) I-V curves of forming process in the devices at different annealing temperatures (300, 350, 400 K); and (**d**) Schematic of oxygen vacancies in IGZO and simple band diagram of the Pd and IGZO layers.

It is known that the O-H bonds in an IGZO thin film before post-annealing react with hydrogen to generate H_2_O molecules when post-annealing is performed under air conditions (Figure 2a,b) [54,55,56]:

(1) $$M-OH+M-OH\to V_{O}{}^{2+}+M-O-M+H_{2}O↑+2e^{-}$$

At this time, H_2_O molecules diffuse and escape, and free electrons (*e*^−^) and oxygen vacancies (*V_O_*^2+^) are created inside IGZO according to Equation (1) [57]. In addition, the interface trap concentration is reduced because the roughness between the SiO_2_ thin film and IGZO is also lowered [58]. The switching and electrical properties of the memristor can be varied by the change inside IGZO before and after post-annealing. A Schottky barrier is formed between Pd and IGZO due to the high work function of Pd (5.3 eV) and SiO_2_ because a high energy barrier is formed as a native oxide between IGZO and p^+^-Si. Therefore, the device needs a forming process to form a current path. When the voltage on the top electrode (V_TE_) is strongly applied, as shown in Figure 2c, the current increases abruptly, and this is called the forming process. Here, O_2_^−^ ions are pushed to the BE, and oxygen vacancies are formed from the TE (Figure 2d). Oxygen vacancies form filaments, which are current paths that are not affected by the Schottky barrier between the TE and IGZO.

The higher the annealing temperature, the lower the voltage generated by the forming process (Figure 2c). In addition, the higher the annealing temperature, the higher the concentration of oxygen vacancies; thus, a filament composed of oxygen vacancies can be formed at a lower electric field. After the forming process, the current characteristics were determined by the SiO_2_ barrier that exists as a native oxide between IGZO and the BE.

**Figure 3:**
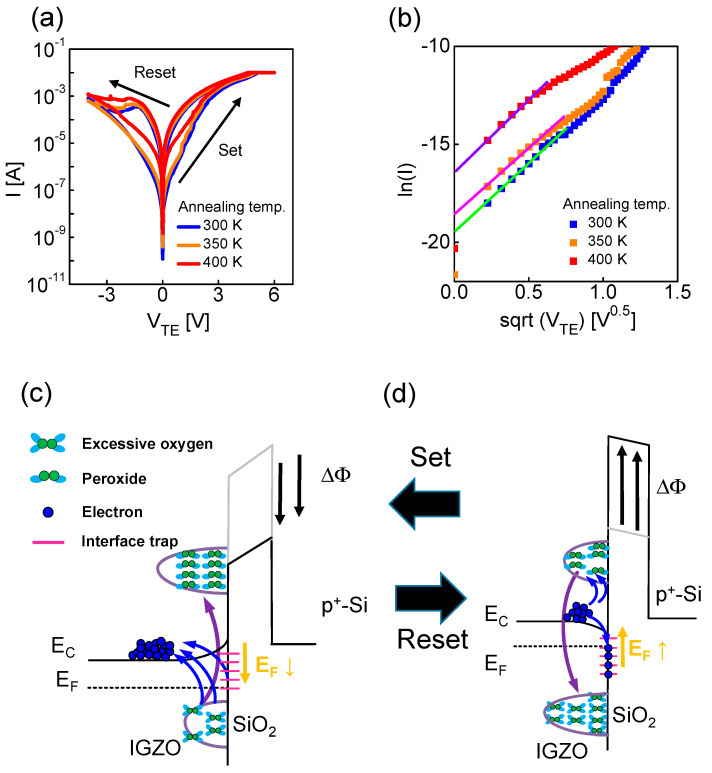
(**a**) I-V curves, including the set and reset processes in the devices with different annealing temperatures (300, 350, 400 K); (**b**) In(I) vs. sqrt(V_TE_) for thermionic emission. EBD of IGZO/SiO_2_/p^+^-Si stack in the (**c**) low-resistance state (LRS) and (**d**) high-resistance state (HRS).

Figure 3a shows the I-V characteristics of the memristor after forming, in which the electrical characteristics are determined by the native oxide. The conduction mechanism of the memristor after forming is attributed to thermionic emission by the native oxide. To prove this, as shown in Figure 3b, I-V curves were converted into ln(I) vs. V^1/2^ as confirmed by the trend with a straight line.

(2) $$I=AA^{*}T^{2}\exp\left[\frac{q}{kT}\left(\sqrt{\frac{qV}{4\pi \varepsilon L_{eff}}}-\Phi_{Bi}\right)\right]$$

The thermionic emission formula is expressed in Equation (2), where *A* is the device area (4 × 10^−4^ cm^2^), A* is the Richardson constant (= 40.8 A/cm^2^K^2^), *T* is the absolute temperature (300 K), ε_IGZO_ is the permittivity of IGZO, *E* is the electric field, *L_eff_* is the effective depletion length, and Φ*_Bi_* is the Schottky barrier height. Figure 3a shows the I-V characteristics of the memristor with different annealing temperatures. The on- and off-currents increased and the on/off ratio decreased with increasing temperature. The higher the post-annealing temperature, the higher the concentration of oxygen vacancies inside the IGZO. Additionally, a positive charge was formed within the IGZO layer, possibly lowering the barrier of the native oxide. The initial barrier can be extracted by the y-intercept (ln(AA*T^2^) − Φ*q*/*kT*)) in the ln(I) vs. sqrt(V_TE_) plot (Figure 3b). The extracted barrier height is 0.69 eV, 0.67 eV, and 0.61 eV at the post-annealing temperatures of 300 K, 350 K, and 400 K, respectively, confirming that the barrier of the initial native oxide decreases with increasing temperature. Next, to explain why the on/off ratio decreases as the temperature increases, first it is necessary to understand the set and reset mechanism of the corresponding memristor. As shown in Figure 3a, a set process with higher conductance occurs when V_TE_ > 0. Conversely, a reset process with lower conductance occurs when V_TE_ < 0. The Fermi level (E_F_) of the IGZO and native oxide interface is lowered during the set process. A reaction occurs in which electrons escape from the trap. The electrons in the interface trap are detrapped, and excessive oxygen reacts with peroxide (Figure 3c):

(3) $$2O^{2-}\Leftrightarrow {\left(O-O\right)}^{2-}+2e^{{}^{-}}$$

The peroxide reaction can be expressed as in Equation (3), and the evidence that this reaction occurs is explained later through the activation energy. The interface charge becomes relatively positive, which lowers the barrier of SiO_cc_ and increases the conductance of the memristor [43,59]. On the other hand, the E_F_ of the IGZO and the native oxide interface is high during the reset process. A reaction in which electrons are injected into the trap occurs. The electrons in the interface trap are trapped, and peroxide reacts with excessive oxygen (Figure 3d). The charge at the IGZO and native oxide interface becomes relatively negative, which increases the barrier of SiO_2_ and lowers the conductance of the memristor. The post-annealing temperature increases, and the oxygen concentration and interface trap concentration decrease. This indicates that the amount of change in the barrier decreases during the switching operation. Therefore, as the post-annealing temperature increases, the on/off ratio decreases. We demonstrate stable DC switching at different temperatures before investing synaptic characteristics in Figure S1.

**Figure 4:**
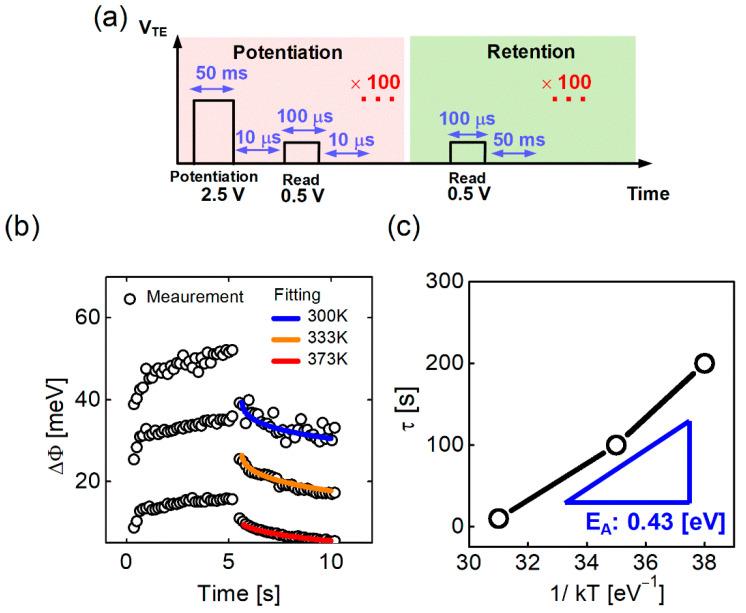
(**a**) Pulse schemes for potentiation and retention; (**b**) Barrier height changes with potentiation and retention pulses; and (**c**) τ vs. 1/kT curve for activation energy.

To understand the mechanism of the switching operation in detail, it is necessary to extract the activation energy required for electron trapping between IGZO and the native oxide. For the extraction of the activation energy, the device was subjected to post-annealing at 300 K, and potentiation and retention experiments were performed at 300 K, 350 K, and 400 K. Figure 4a shows the pulse schematic used for the potentiation and retention experiments. For potentiation, a pulse with a magnitude of 2.5 V and a pulse width of 50 ms was applied 100 times to make the resistance of the memristor sufficiently low (Figure 4a, left). A pulse with a pulse size of 0.5 V and a pulse width of 100 μs was applied for the reading. In the potentiation operation, the interval between the read pulse and the potentiation pulse is very short (10 μs) to retain the short-term memory component during the read operation. The interval between the reading pulses in the retention operation is 50 ms. This was applied 100 times, and the retention characteristics were monitored for 5 s (Figure 4a, right). The current extracted by the read pulse was converted to Φ using Equation (1). The amount of change (ΔΦ) in the potentiation and retention periods, as compared with the barrier (Φ) at 0 s, is shown in Figure 4b. τ was extracted by fitting ΔΦ obtained in the retention test for each temperature, and the activation energy (0.43 eV) was extracted using the slope in the τ vs. 1/kT curve (Figure 4c) [59,60]. This was confirmed as the activation energy of the peroxide reaction in previous studies [61,62].

**Figure 5:**
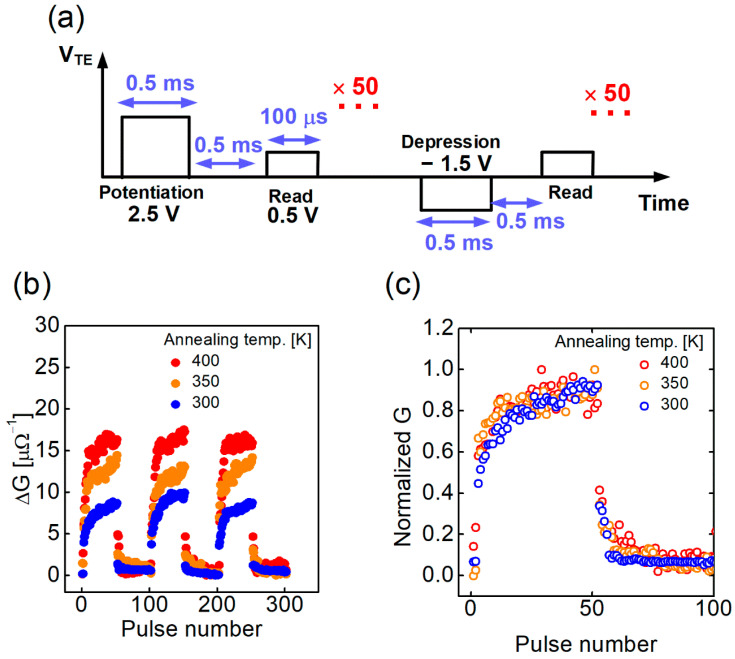
(**a**) Pulse schemes of potentiation and depression; (**b**) Potentiation and depression of devices with different annealing temperatures (300, 350, 400 K); and (**c**) Potentiation and depression curves after normalization of conductance.

**Figure 6:**
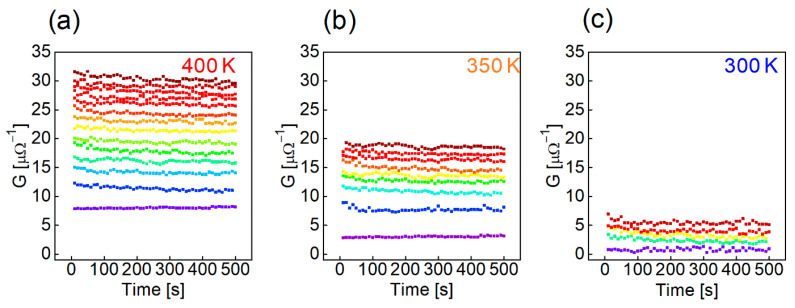
Short retention test of multi-level states in the devices with different annealing temperatures: (**a**) 300 K, (**b**) 350 K, and (**c**) 400 K.

**Figure 7:**
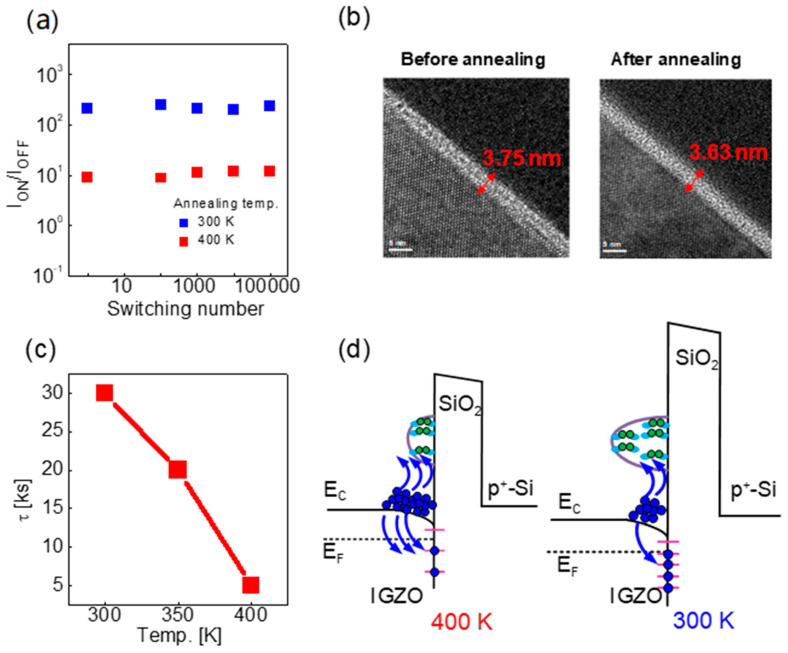
(**a**) On/off ratio as a function of switching number in devices based on annealing temperature (300 K and 400 K); (**b**) TEM images of samples before and after annealing; (**c**) τ vs. annealing temperature; and (**d**) EBD, including oxygen vacancies and trapping of electrons for the explanation of retention.

Figure 5a shows the pulse sequence used to investigate the post-annealing effect with different temperatures on the potentiation and depression characteristics. For potentiation, 50 pulses with a magnitude of 2 V and a pulse width of 0.5 ms were applied 50 times, and for depression, pulses with a magnitude of −1.5 V and a pulse width of 0.5 ms were applied 50 times. For the reading, 0.5 V and a pulse width of 100 μs were applied. It was found that the higher the annealing temperature, the greater the change rate of conductance, as shown in Figure 5b. The higher the annealing temperature, the lower the initial barrier, indicating that the conductance is sensitively changed, even with a small barrier change. Therefore, when the post-annealing temperature is high, the change in conductance is large even though the change in barrier during the set/reset operation is low. Also, as shown in Figure 5c, the linearity is not much different regardless of the post-annealing temperature when the maximum value of conductance in the potentiation/depression result is normalized to 1 (Figure 5c). The reason that the linearity is constant regardless of the post-annealing temperature is explained in the next sections, along with the retention characteristics. Figure 6a–c shows multiple conductance states by adjusting the number of potentiation pulses at 300 K, 350 K, and 400 K, respectively. During the read operation, the read pulse scheme was the same as that in Figure 5a, and the interval between reading pulses was 10 s, applied 50 times. More conductance states can be created during the potentiation operation when the amount of change in the conductance is large. It is important to ensure a margin between the conductance states considering the conductance variation over time when used in actual applications [25]. Finally, through the experiment, 5, 9, and 14 states were demonstrated with different post-annealing temperatures of 300 K, 350 K, and 400 K, respectively. This has a great effect on the neural network characteristics, and we discuss the conductance state number and the neural network performance and correlation in the following sections. The endurance with the on/off ratio was determined as a function of the switching number in which one cycle was one potentiation/depression pair, as shown in Figure 7a. It was confirmed that the on/off ratio did not change significantly during the 100,000 cycles at 300 K and 400 K.

In Figure 7b, the thickness of SiO_2_ based on the annealing temperature in the TEM image is the same. The endurance can be determined by the thickness of SiO_2_. The thicker the SiO_2_ layer, the longer the device can be switched. As shown in the TEM image (Figure 7b), the annealing process did not affect the thickness of SiO_2_. Therefore, the endurance characteristics were not affected by the annealing temperature, and the device demonstrated good endurance characteristics because IGZO was deposited under high oxygen conditions [50]. On the other hand, the higher the post-annealing temperature, the shorter the retention time (Figure 7c). In addition, the higher the post-annealing temperature, the higher the number of oxygen vacancies inside IGZO and the higher the concentration of free electrons. Therefore, it increases the electron trapping probability at the IGZO interface (Figure 7d).

Two factors can affect the linearity of potentiation. The first factor is the change in the amount of voltage distribution between the switching layer and the bulk and the parasitic resistance during potentiation operation, as mentioned in [50]. The resistance of the switching layer is gradually decreased when potentiation is performed by pulses under the same conditions. Therefore, the voltage applied to the switching layer is gradually decreased. At this time, the larger the change in the conductance during potentiation operation, the faster the voltage applied to the switching layer decreases. This process accelerates conductance saturation by potentiation and adversely affects the linearity of the conductance change by pulse number. That is, the first factor at the post-annealing temperature of 400 K adversely affects the linearity of the potentiation. Second, retention characteristics could affect the linearity of potentiation. The conductance decreases during the reading interval time between potentiation pulses. The decrease in conductance is accelerated during the interval time when the retention characteristics are not good, which prevents the conductance saturation of potentiation. Therefore, linearity can be improved due to poor retention. The second factor is that the post-annealing temperature of 400 K in our experimental sample has a good effect on the linearity. The first and the second factors are compensated; thus, the linearity characteristics of potentiation are not that different at different annealing temperatures. However, the depression linearity is not good for all samples. Oxygen with a negative charge drifts toward the BE side since depression occurs when a negative bias is applied to V_TE_. Therefore, the oxygen concentration on the BE side is high, and the reaction between peroxide and excessive oxygen occurs faster than potentiation, indicating that the linearity of depression is not good.

**Figure 8:**
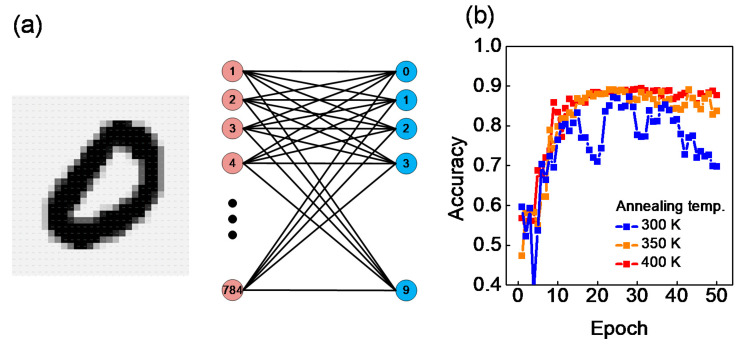
Deep neural network simulation using MNIST: (**a**) Single-layer network including input and output nodes; (**b**) Accuracy as a function of epoch for devices with different annealing temperatures (300, 350, 400 K).

A deep neural network (NN) was virtually implemented using MATLAB, and MNIST simulation was performed using the network (Figure 8a). The DNN includes 784 input nodes for the data of a picture of a handwritten number with 28 × 28 pixels and 10 output nodes to represent the correct answer to the number as an output value. Here, the input and output nodes were directly connected without a hidden layer. The characteristics of the IGZO memristors with different post-annealing temperatures were applied to the biological synapses of the DNN. The linearity and number of conductance states were introduced to the DNN, where the weight characteristics of the synapses, shown in Figure 5c and Figure 6, can affect the accuracy of the MNIST pattern recognition. The weight values of the synapses range between 0 and 1.

(4) $$1-\exp\left(-\left(N\right)/A_{P}\right)$$

(5) $$\exp\left(-\left(N\right)/A_{D}\right)$$

The change in weight for the potentiation/depression pulse was used in the simulation after fitting the normalized potentiation/depression curve of Figure 5c with Equations (4) and (5). *N* is the pulse number, and A_P_ and A_D_ in Equations (4) and (5) are fitting parameters. Fitting was performed by adjusting the A_P_ of Equation (4) for potentiation and the A_D_ of Equation (5) for depression. The A_P_ and A_D_ values for all samples were equally set to 0.2 and 0.1, respectively, because the linearity, regardless of the post-annealing temperature, is no different. The experimentally obtained values were used for the number of conductance states in the simulation. The recognition rate was calculated by performing a test with 10,000 data after training with 50,000 data per 1 epoch. We confirmed the most suitable annealing temperature conditions for the application through the above simulation. The memristor baked at a temperature of 300 K had the lowest conductance state of 5, so the recognition rate is the lowest, and the recognition rate was not stable because the weight easily changed even after much training (Figure 8b). However, the memristor annealed at a temperature of 400 K showed the highest and most stable recognition rate because it had the highest conductance state of 14 (Figure 8b).

## 4. Conclusions

In this work, post-annealing treatment was conducted to control the synaptic characteristics in Pd/IGZO/SiO_2_/p^+^-Si memristor devices. The Schottky barrier between the top electrode and IGZO layers adjusts the conductance in the memristor system. It was found that the annealing temperature of the devices can affect the barrier height, on/off ratio, activation energy, and potentiation/depression characteristics. Moreover, we investigated the linearity of potentiation and depression in the devices at different annealing temperatures. Finally, the accuracy of MNIST in the neural network was calculated considering the annealing temperature of the device.

## Data Availability

Not applicable.

## Supplementary Materials

The following supporting information can be downloaded at: https://www.mdpi.com/article/10.3390/nano12203582/s1, Figure S1: DC 100 cycles of the device at different temperatures (a) 300 K, (b) 350 K, and (c) 400 K.
