# Supplementary material for: Effect of Post-Annealing on Barrier Modulations in Pd/IGZO/SiO2/p+-Si Memristors
Source: Nanomaterials (Basel). 2022 Oct 13;12(20):3582. doi: 10.3390/nano12203582 (PMC9610976; doi:10.3390/nano12203582)
Supplement: Supplementary file 1 [file nanomaterials-12-03582-s001.zip › nanomaterials-1951486-supplementary.pdf]

Supplementary Information

Effect of Post-Annealing on Barrier Modulations in Pd/IGZO/SiO_2/p^+-Si Memristors

Donguk Kim [1,†], Hee Jun Lee [1,†], Tae Jun Yang [1], Woo Sik Choi [1], Changwook Kim [1], Sung-Jin Choi [1], Jong-Ho Bae [1], Dong Myong Kim [1], Sungjun Kim [2,*] and Dae Hwan Kim [1,*]

[1] School of Electrical Engineering, Kookmin University, Seoul 02707, Korea

[2] Division of Electronics and Electrical Engineering, Dongguk University, Seoul 04620, Korea

[*] Correspondences: sungjun@dongguk.edu (S.K.); drlife@kookmin.ac.kr (D.H.K.)

[†] They equally contributed to this work.

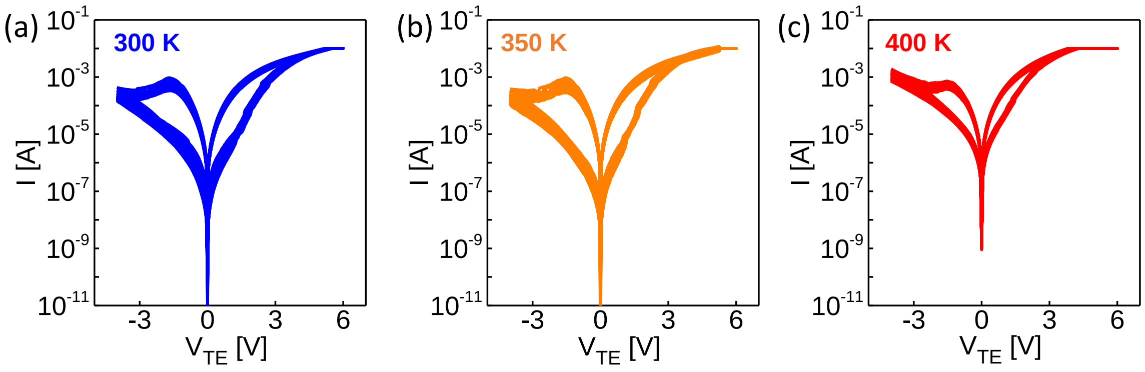

Figure S1. DC 100 cycles of the device at different temperatures (a) 300 K, (b) 350 K, and (c) 400 K.
